# Natural Products as Targeted Modulators of the Immune System

**DOI:** 10.1155/2018/7862782

**Published:** 2018-11-14

**Authors:** Kai Wang, Michael Conlon, Wenkai Ren, Bill B. Chen, Tomasz Bączek

**Affiliations:** ^1^Institute of Apicultural Research, Chinese Academy of Agricultural Sciences, Beijing, China; ^2^CSIRO Health and Biosecurity, Adelaide, Australia; ^3^Guangdong Provincial Key Laboratory of Animal Nutrition Control, Institute of Subtropical Animal Nutrition and Feed, College of Animal Science, South China Agricultural University, Guangzhou, China; ^4^Jiangsu Co-Innovation Center for Important Animal Infectious Diseases and Zoonoses, Joint International Research Laboratory of Agriculture and Agri-Product Safety of Ministry of Education of China, College of Veterinary Medicine, Yangzhou University, Yangzhou, China; ^5^University of Pittsburgh School of Medicine, Pittsburgh, USA; ^6^Department of Pharmaceutical Chemistry, Medical University of Gdańsk, Gdańsk, Poland

For decades, compounds derived from natural products have demonstrated their effectiveness as therapeutic agents in different areas, such as metabolic disorder, cardiovascular diseases, inflammation, and neurological disorders. Natural products, like herbal medicines, fatty acids, and probiotics, also are implicated in the regulation of immune function. They control the immune system in a pleiotropic manner and participate in various processes of the adaptive/innate immunity. Therefore, natural products have great potential for targeted immune modulators, in the treatment of certain types of immunologic and inflammatory diseases, like rheumatoid arthritis, plaque psoriasis, ankylosing spondylitis, Crohn's disease, and ulcerative colitis.

This special issue involves 12 original papers and 2 review papers selected by the editors which provides our readers a deeper understanding for using natural products as targeted modulators of the immune system. These papers are summarized as follows.

Polyphenols are pharmacologically active natural products with well-known immunomodulatory activities. S. Ding et al. reviewed the immune-regulating properties of polyphenols from the perspectives of molecular immunity and epigenetic inheritance. They also raised several recommendations for the development of polyphenols as immune factors, including improving polyphenols' bioavailability in the host, appropriately determining different immunological responses by polyphenols, and getting a comprehensive understanding of synergistic effects by the polyphenols. In this special issue, the immune-modulating effect of two typical natural polyphenols, luteolin and curcumin, has been investigated. Y. Liao et al. studied the effects of luteolin on apoptosis or autophagy in the macrophages. They noticed that luteolin effectively improved ANA-1 macrophage apoptosis and autophagy via regulating the p38, ERK, and Akt pathways to inhibit the expression of antiapoptotic proteins (Bcl-2 and Beclin-1) and increase the antiapoptotic protein expressions (caspase-3 and caspase-8). T. Larussa et al. uncovered that curcumin changed the expressions of interleukin- (IL-) 17 and indoleamine 2,3-dioxygenase (IDO) during *Helicobacter pylori*-infected human gastric mucosa, which provides us novel understanding into the knowledge of the immune pathways.

Innate immunity plays key roles against pathogens invading, and pattern recognition receptor (PRR) is known as a fundamental immune receptor for the innate immune system. In this review, H.-Y. Guo et al. summarized the functions of two PRRs, Toll-like receptors (TLRs) and retinoic acid-inducible gene I- (RIG-I-) like receptors (RLRs), in resisting flavivirus invasion. Moreover, M. Rodríguez-Valentín et al. using a natural *Coriolus versicolor*-derived polysaccharide peptide (PSP) demonstrated its anti-HIV properties, via decreasing viral replication and promoting the releases of antiviral chemokines in THP1 cells and human PBMCs. They further noticed that these effects were TLR4-dependent, since TLR4 inhibition caused counter effects in chemokine expression and HIV-1 replication in PSP-treated cells.

In recent years, use of medicinal plants is gaining wide recognition due to their versatility, safety, and cost-effectiveness. *Fagonia indica* is a traditional medical plant in India which has been studied and practiced against a broad range of diseases. F. Azam et al. studied the hepatoprotective activity of *F. indica* extract using a mouse liver injury model induced by thioacetamide. They showed that *F. indica* extract administration ameliorated serological and histological changes in the mice liver. This extract also altered expression of proinflammatory and hepatic markers in the liver, suggesting a promising immune regulatory role for the plant in curing liver injury. Propolis is a natural product originally produced by honeybees (*Apis mellifera*) from tree buds. L. Sun et al. fractionalized Chinese propolis with different solvents and compared their antinociceptive effects using rodent models. They found that the antinociceptive activities from Chinese propolis fractions are different, which relate to their flavonoid compositions. As the main source of Chinese propolis in the northern China temperate zone is poplar buds, S. Peng et al. investigated the beneficial effects of poplar buds in type-2 diabetic mice induced by streptozotocin. They showed that poplar bud extracts decreased blood glucose levels and insulin resistance and significantly relieved dyslipidemia, oxidative stress, and inflammation in type-2 diabetes mice.

This special issue also contains work using naturally derived products from various sources, including functional lipids, antioxidants, and bile acid derivatives, as well as natural glucosides. Tributyrin is a structured lipid with three butyrate molecules esterified to glycerol. B. Glueck et al. showed the protective effects of tributyrin supplementation following chronic-binge ethanol exposure in mice, which was linked to the modulation on the gut innate immune responses and restored oxidative stress. They found a possible involvement of tributyrin in maintaining intestinal integrity in this experiment. Thioredoxin reductase (TrxR1) is known as an antioxidant enzyme which helps defense against oxidative stress. F. Chen et al. found that a miRNA-mediated posttranscriptional mechanism is involved in H_2_O_2_-induced TrxR1 expression in endothelial cells, suggesting an important role of miRNAs in response to oxidative stress and immune dysfunctions. On the other hand, S. Zhao et al. noticed that secondary bile acid deoxycholic acid (DCA), which is considered to be associated with the development of inflammatory bowel disease (IBD). Even though they showed that DCA enema aggravated DSS-induced mice colitis, their findings suggest that trigger inflammasome activation by DCA via S1PR2 may represent a novel therapeutic target for IBD management. Inflammasome activation in the eyes also has been linked to the pathogenesis of age-related macular degeneration. X. Jin et al. investigated the protective effects of cyanidin-3-glucoside against 4-hydroxyhexenal-induced inflammatory damages in human retinal pigment epithelial cells. Cyanidin-3-glucoside showed potent inhibitive effects on NLRP3 inflammasome activation hallmarks induced by HHE, which are associated with decreased JNK activation.

Taken together, this special issue provides us novel insights for the prevention and treatment of immune-related diseases using natural products.

